# Role and prevalence of antibiosis and the related resistance genes in the environment

**DOI:** 10.3389/fmicb.2014.00520

**Published:** 2014-10-02

**Authors:** Sylvie Nazaret, Rustam Aminov

**Affiliations:** ^1^UMR 5557 Ecologie Microbienne, CNRS, Ecole Nationale Vétérinaire de Lyon, and Université Lyon 1, Université de LyonVilleurbanne, France; ^2^Section for Bacteriology, Pathology and Parasitology, National Veterinary Institute, Technical University of DenmarkFrederiksberg C, Denmark

**Keywords:** antibiotic resistance, mobile genetic elements, environment, model

It becomes increasingly clear that the basis of antibiotic resistance problem among bacterial pathogens is not confined to the borders of clinical microbiology but has broader ecological and evolutionary associations. This Research Topic “Role and prevalence of antibiosis and the related resistance genes in the environment” in *Frontiers in Microbiology: Antimicrobials, Resistance, and Chemotherapy* presents the examples of occurrence and diversity of antibiotic resistance genes (ARGs) in the wide range of environments, from the grasslands of the Colombian Andes, to the dairy farms and small animal veterinary hospitals in the United Stated, and to the various environments of Continental Europe and Indochina. Besides, various genetic mechanisms and selection/co-selection factors contributing to the dissemination and maintenance of ARGs are presented. The topic is finalized by the mathematical modeling approach to access the probability of rare horizontal gene transfer (HGT) events in bacterial populations.

The opinion article by Martínez (2012) summarizes our present understanding of the cycle of ARGs acquisition by bacterial pathogens. The environmental microbiota harbors a vast diversity of genes, which we usually classify as conferring resistance to antibiotics. In natural ecosystems, however, their role may be different and not necessarily associated with this function. Yet, if the certain metabolic genes are acquired by commensal/pathogenic microbiota and appeared to be conferring selective advantage under the pressure of antibiotics, their primary function under these new ecological circumstances becomes resistance to antibiotics. Moreover, upon the amplification under the antibiotic selective pressure, these ARGs are released into the environment thus contributing to the rise of antibiotic resistance in other ecological compartments.

Evidence for the environmental contamination by ARGs can be seen in several articles of this Research Topic. For example, despite the low antibiotic usage in the grassland farms located in the Colombian Andes, there is a significant diversity of tetracycline resistance genes in the microbiota of the animal gut and the environment (Santamaría et al., 2011). But the diversity of the *tet* genes in the former ecosystem is higher thus suggesting the gene flow from the animals into the environment. Another study involved the isolation and characterization of the CTX-M [a major type of extended-spectrum beta-lactamase (ESBL)] producing *Escherichia coli* strains from soils, cattle, and the farm environment in the Burgundy region of France (Hartmann et al., 2012). Environmental and animal strains appeared to be clonally related. The study also suggests a long-term survival of the CTX-M-producing *E. coli* strains in soil since the last manure application has been done 1 year before the actual sampling. Czekalski et al. (2012) demonstrated the increased levels of multidrug-resistant bacteria and ARGs in Lake Geneva, Switzerland due to the discharge from the local wastewater treatment plant. Counterintuitively, wastewater treatment resulted in selection of extremely multidrug-resistant bacteria and accumulation of ARGs although the total bacterial load was substantially decreased. A less favorable situation with the treatment of wastewater is in Indochina, which includes Vietnam, Thailand, Cambodia, Lao PDR, and Myanmar. Suzuki and Hoa (2012) summarized the current knowledge regarding the presence of quinolones, sulfonamides, and tetracyclines as well as the corresponding ARGs in this region. They concluded that: (1) no correlation exists between the quinolone contamination and quinolone resistance; (2) occurrence of the *sul* sulfonamide resistance gene varies geographically; and (3) microbial diversity relates to the oxytetracycline resistance level.

Thames et al. (2012) used qPCR to investigate the effect of feeding milk replacers with various antibiotic doses on the excretion of ARGs by dairy calves. Interestingly, no significant differences have been found in the absolute numbers of ARGs excreted. After the normalization to the 16S rRNA genes the relative *tet*(O) concentration appeared to be higher in animals fed the highest therapeutic doses of antibiotic. Besides, antibiotic feeding provided no obvious health benefits. The authors concluded that the greater than conventional nutritional intake in the study outweighs the previously reported health benefits of antibiotics. Ghosh et al. (2012) reported an interesting observation regarding the carriage of multi-drug resistant enterococci by resident cats in small animal veterinary hospitals. Genotypically identical strains were isolated from cats and surfaces of cage door, thermometer, and stethoscope suggesting that the animals may be involved in cross-contamination of the hospital environment.

What are the factors supporting the dissemination of ARGs? Among the genetic mechanisms, Heuer et al. (2012) identified IncP-1ε plasmids as important vectors for horizontal transfer of antibiotic resistance in agricultural systems. These plasmids are transferable to a wide range of *Beta*- and *Gammaproteobacteria*, with the concurrent transfer of ARGs. Stalder et al. (2012) extensively reviewed the role of integrons in the environmental spread of antibiotic resistance. The main conclusion is that many stress factors including but not limited to antibiotics, quaternary ammonium compounds or high concentrations of heavy metals result in selection of class 1 mobile integron-harboring bacteria. Consistent with the findings of Czekalski et al. (2012), wastewater treatment plants may serve as hot spots for class 1 mobile integron dissemination, with the concurrent dissemination of ARGs. Seiler and Berendonk (2012) reviewed the role of heavy metals in co-selection of antibiotic resistance in soil and water bodies impacted by agriculture and aquaculture. The metals that are extensively used in agriculture such as copper and zinc have the potential to co-select for antibiotic resistance in the environment.

Conventional experimental design for identification of HGT events usually relies on the detection of initial transfer occurrences in fairly small bacterial populations. This approach, however, may fail to detect the rare transfer events thus leading to an inappropriate conclusion regarding the long-term HGT effects. Townsend et al. (2012) addressed this problem using the models that take into consideration various degrees of natural selection, growth dynamics of bacteria with differing fitness, genetic drift, and other variables to build a probabilistic framework for detection of HGT within a given sampling design. This approach will assist to a better design of experiments aimed at detection and analysis of HGT in natural setting.

In summary, the papers in this Research Topic contribute to a better understanding of the dynamic of ARGs within and among different ecological compartments. They emphasize the need for a careful monitoring of the release of pre-selected ARGs into the environment from which they may enter the human food chain. Especially worrying are the findings that the current wastewater treatment systems may serve as hot spots for the amplification of multidrug-resistant bacteria, ARGs, and mobile genetic elements. The topic calls for more research that is needed for identification of crucial checkpoints to limit the circulation of ARGs among different ecological compartments.

## Conflict of interest statement

The authors declare that the research was conducted in the absence of any commercial or financial relationships that could be construed as a potential conflict of interest.
